# Spindle and kinetochore associated complex subunit 1 regulates the proliferation of oral adenosquamous carcinoma CAL-27 cells *in vitro*

**DOI:** 10.1186/1475-2867-13-83

**Published:** 2013-08-20

**Authors:** Bin Zhang, Ke Yi Li, Hai Ying Chen, Shao Dong Pan, Li Cheng Jiang, Ya Ping Wu, Shu Wei Liu

**Affiliations:** 1 Department of Anatomy, Shandong University, School of Medicine, Jinan, Shandong 250012, P. R. China; 2 Department of Oral and Maxillofacial Surgery, Liaocheng People’s Hospital, Liaocheng, Shandong 252000, P. R. China; 3 Key Laboratory of Oral and Maxillofacial-Head and Neck Medicine, Central Laboratory, Liaocheng People’s Hospital and Liaocheng Clinical School, Liaocheng, Shandong 252000, P. R. China; 4 Department of Clinical Chemistry and Haematology, University Medical Centre Utrecht, G03.550, Heidelberglaan 100, 3584 CX, Utrecht, The Netherlands

**Keywords:** Oral adenosquamous carcinoma, CAL-27 cells, Cell proliferation, SKA1, RNAi

## Abstract

**Background:**

The prognosis of oral squamous cell carcinoma is very poor due to local recurrence and metastasis. This study explores the molecular events involved in oral carcinoma with the goal of developing novel therapeutic strategies. The mitotic spindle is a complex mechanical apparatus required for the accurate segregation of sister chromosomes during mitosis. Spindle and kinetochore associated complex subunit 1 (SKA1) is a microtubule-binding subcomplex of the outer kinetochore that is essential for proper chromosome segregation. In recent years, much attention has been focused on determining how SKA proteins interact with each other, as well as their biological role in cancer cells. However, the precise role of SKA1 in oral carcinoma remains unknown.

**Methods:**

In order to investigate the role of SKA1 in oral cancer, we employed lentivirus-mediated shRNA to silence SKA1 expression in the CAL-27 human oral adenosquamous carcinoma cell line.

**Results:**

Depletion of SKA1 in CAL-27 cells significantly decreased cell proliferation, as determined by MTT and colony formation assays. These results strongly demonstrate that reduced SKA1 protein levels may cause inhibition of tumor formation. The shRNA-mediated depletion of SKA1 also led to G2/M phase cell cycle arrest and apoptosis.

**Conclusion:**

This is the first report to show that SKA1 plays an important role in the progression of oral adenosqamous carcinoma. Thus, silencing of SKA1 by RNAi might be a potential therapy for this disease.

## Background

Worldwide, there are an estimated 405,000 new cases of oral cancer diagnosed each year [1]. Despite advances in the therapeutic management of squamous cell carcinoma over the last few decades, the prognosis is still very poor due to local recurrence and metastasis [2-4]. This disappointing outcome, even with existing therapies, strongly suggests that novel targeted therapeutic agents are needed to improve the treatment of patients diagnosed with oral cancer. The successful use of small interfering RNA (RNAi) to downregulate gene expression in several model systems has led to many attempts to explore this methodology in cancer therapeutic settings [5]. RNAi is a post-transcriptional mechanism of gene silencing through chromatin remodeling, inhibition of protein translation, or direct mRNA degradation, which is ubiquitous in eukaryotic cells [6]. Along these lines, several recent studies have documented the successful downregulation of gene expression, resulting in increased apoptosis and decreased proliferation in many cancer cell lines [4,7]. Long-lasting RNAi-based gene silencing can be achieved using lentivirus-based expression systems, which drive the production of short hairpin RNAs (shRNAs) [8]. Lentiviral vectors have become a promising tool for the establishment of transgenic animals and manipulation of the mammalian genome [9].

Mitotic chromosome segregation requires kinetochores to generate physical connections between chromosomes and spindle microtubule polymers [10]. Notably, spindle and kinetochore-associated protein complexes have several key properties, and play an important role in coupling chromosome movement to microtubule depolymerization [11,12]. Spindle and kinetochore associated complex subunit 1 (SKA1) is a microtubule-binding subcomplex of the outer kinetochore that is essential for proper chromosome segregation [13,14]. Depletion of SKA1 proteins results in sparse microtubule arrays that often exhibited twisted or bent spindles. The chromosomes appear to interact with the spindle microtubule, but fail to organize into metaphase plates [15,16]. Much attention has been focused in recent years on how SKA proteins interact with each other, and their biological role in cancer cells. However, until now, the precise role of SKA1 in oral carcinoma has remained unclear. Therefore, the goal of this study was to determine if SKA1 plays a significant role in oral adenosquamous cell carcinoma by using lentivirus-mediated shRNA for the functional gene knockdown of SKA1 expression in CAL-27 cells.

## Results and discussion

### Method validation

**Figure 1:**
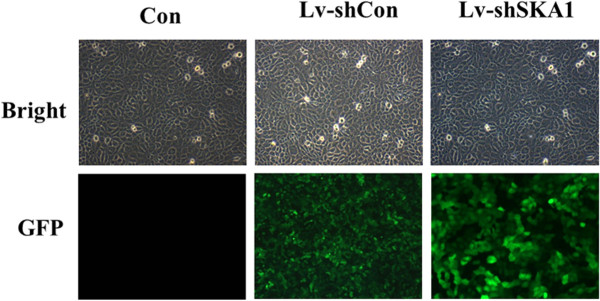
**Infection of cells with lentiviral shRNA targeting SKA1 in CAL-27 cells.** GFP expression in uninfected (Con), Lv-shSKA1-infected, and Lv-shCon-infected CAL-27 cells indicates lentivirus infection efficiency in CAL-27 cells. Transduction efficiency was estimated 6 days after infection at MOI of 10. Light micrograph (left); Fluorescent micrograph (right) (200×).

We used recombinant lentivirus harboring shRNA for SKA1 to infect CAL-27 cells. The successful infection and efficiency were confirmed by evaluating GFP expression levels. After infection, approximately 90% of cells infected with Lv-shSKA1 expressed GFP (Figure 1).

**Figure 2:**
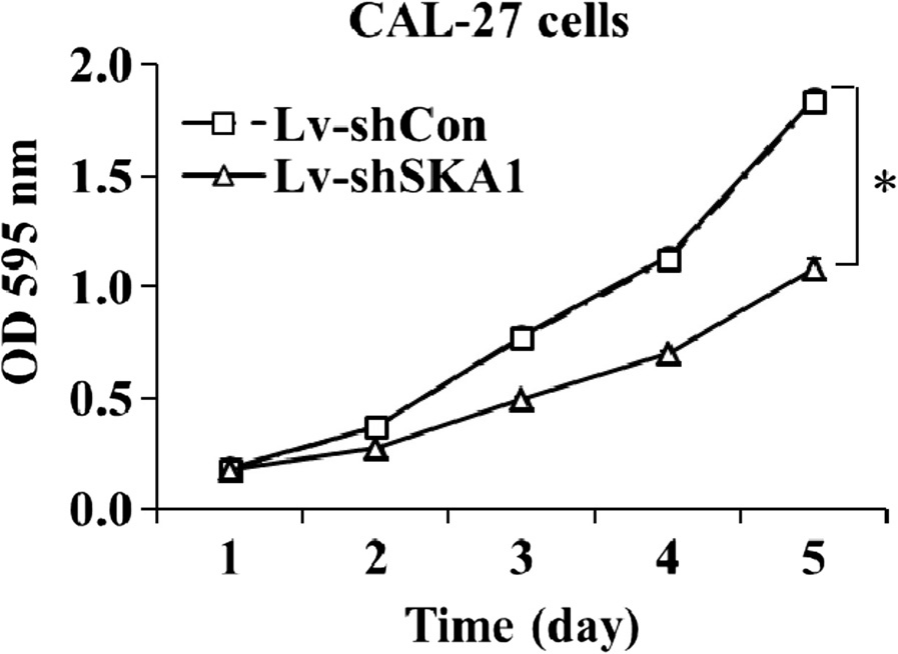
**Western blot analysis of SKA1 protein expression levels in Lv-shSKA1-infected, LV-shCon-infected and uninfected CAL-27 cells (Con).** *p < 0.01. Data are representative of three independent experiments.

These results suggest that the lentivirus infections are successful and highly efficient. The effect of Lv-shSKA1 on SKA1 expression in CAL-27 cells was observed by western blot analysis (Figure 2).

Western blot analysis showed that SKA1 protein was not expressed in Lv-shSKA1 -infected cells. In contrast, equal protein expression was observed in uninfected cells and Lv-shCon-infected (negative control) cells. Therefore, Lv-shSKA1 specifically and strongly abolished SKA1 expression in CAL-27 cells.

### SKA1 affects CAL-27 cell proliferation

**Figure 3:**
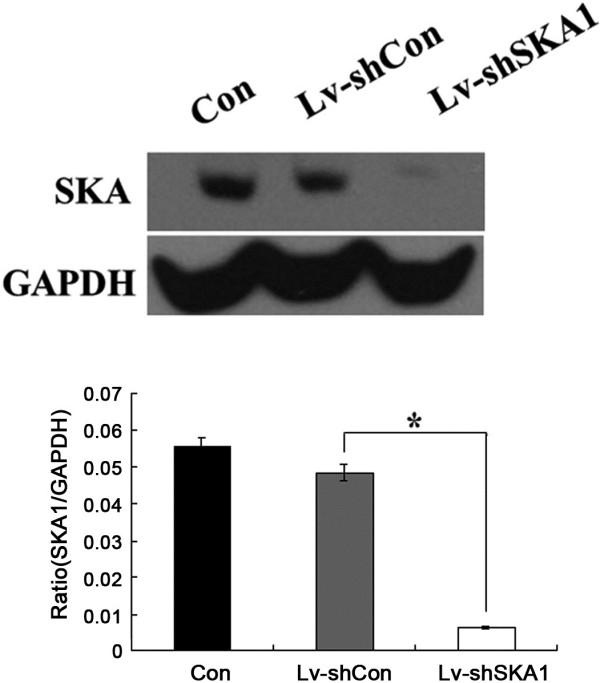
**SKA1 affects the proliferation of CAL-27 cells.** CAL-27 cells were seeded into 96-well plates at a density of 2.5 × 10^3^ cells/well in triplicate, and proliferation rates were measured by the MTT assay. Data are representative of three independent experiments. *p < 0.01.

To elucidate the functional role of SKA1 in oral adenosquamous carcinoma cells, we examined the effect of SKA1 knockdown on the proliferation and colony formation of CAL-27 cells. Cell proliferation was evaluated by the MTT assay, and the results showed that downregulation of SKA1 expression in CAL-27 cells significantly decreased cell viability. Five days post-infection, the viability of Lv-shSKA1-infected CAL-27 cells decreased compared to Lv-shCon-infected cells (*p* < 0.01; Figure 3). Cell viability was the same in Lv-shCon-infected and uninfected CAL-27 cells.

### Downregulation of SKA1 in CAL-27 cells significantly reduces colony formation

**Figure 4:**
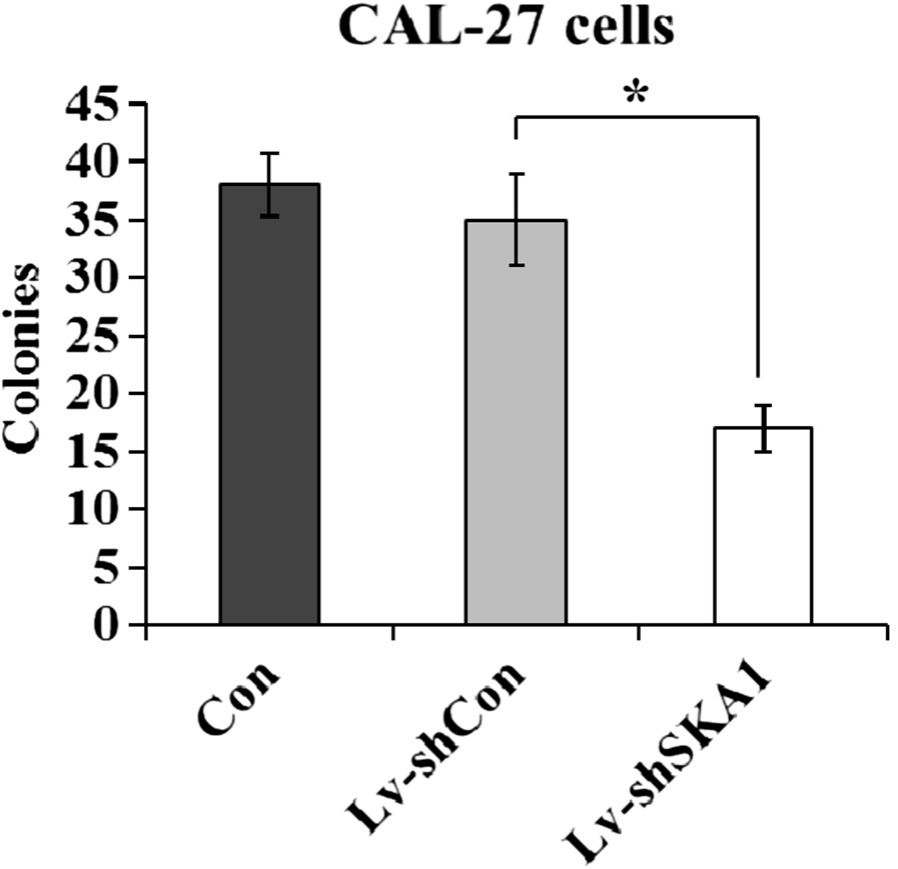
**SKA1 affects the colony formation of CAL-27 cells.** After 13 days, cells were stained with Giemsa to assess colony formation rates. Quantification of colony formation rates in uninfected, Lv-shSKA1-infected, and Lv-shCon-infected CAL-27 cells are shown as histograms. Data are representative of three independent experiments. Error bars represent standard deviation (SD), *p < 0.01.

**Figure 5:**
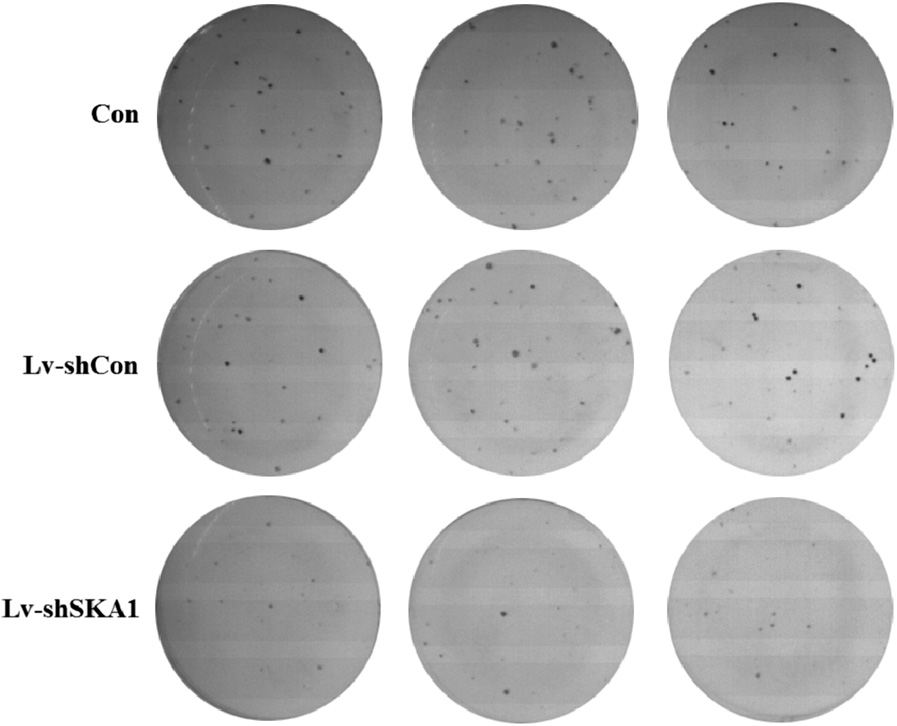
**SKA1 affects the colony formation of CAL-27 cells.** Colonies stained with Giemsa were observed by light microscopy (200×).

To determine the role of SKA1 in human oral adenosquamous tumorigenesis *in vitro*, we analyzed CAL-27 tumor cell colony formation in a soft agar assay. Downregulation of SKA1 in CAL-27 cells caused a substantial reduction in colony formation (Figures 4 and 5) compared with uninfected and Lv-shCon-infected cells (*p* < 0.01).

Results from the colony formation assay strongly indicate that reduced SKA1 protein levels might result in significant inhibition of tumor formation in human oral adenosquamous cancer cells. Furthermore, the data showed that SKA1 was critical for the proliferation and tumorigenicity of human oral adenosquamous cancer cells *in vitro*.

### SKA1 depletion affects the cell cycle of CAL-27 cells

Flow cytometry was used to determine the effect of Lv-shSKA1 infection on the cell cycle of CAL-27 cells. As shown in Table 1, down-regulation of SKA1 led to an arrest in G 1/G 0 and G 2/M phase, the percentage of cells in S phase reduced and sub-G 1 appeared. This cell cycle analysis result suggesting the sequential events of cell cycle arrest by SKA1 silencing in CAL-27 cells is followed by apoptosis process. However, there were no significant differences in cell cycle distribution between in noninfected (Con) group and Lv-shCon group.

**Table 1:** SKA1 depletion affects the cell cycle of CAL-27 cells

**Phase**	**G0/G1**	**S**	**G2/M**	**Sub-G1**
Con	59.03 ± 1.59	29.53 ± 2.66	11.40 ± 1.04	0.45 ± 0.06
Lv-shCon	56.33 ± 0.38	31.60 ± 0.78	12.07 ± 0.75	0.43 ± 0.06
Lv-shSKA1	61.73 ± 1.53**	19.27 ± 1.52	19.03 ± 0.49*	1.00 ± 0.06*

The flow cytometric analysis of cell cycle of uninfected CAL-27 cells (Con), Lv-shSKA1, and Lv-shCon. The percentages of the cells in each phase of the cycle are presented as the mean ± SD of three independent experiments. *represents p < 0.05 between Lv-shSKA1group and Lv-shCon group. **represents p < 0.01 between Lv-shSKA1group and Lv-shCon group. There were no significant differences in cell cycle distribution between noninfected (Con) group and Lv-shCon group (p > 0.05).

## Discussion

Upon entry into mitosis, the microtubule network is rearranged to form the mitotic spindle, which results in segregation of sister chromatids [17-19]. Central to this process is the proper attachment of spindle microtubules to kinetochores, proteinaceous structures assembled on centromeric chromatin [20]. Three subunits of the SKA1 complex (SKA1, SKA2, and SKA3/RAMA) are direct components of the kinetochore-microtubule interface [18]. SKA2 protein requires SKA1, a protein-binding partner, for stability, and SKA2 is required for the correct assembly of SKA1 on the kinetochore [20]. It is well established that depletion of the SKA complex leads to apoptosis in many mammalian cell lines. Here, we evaluated the effect of SKA1 depletion in CAL-27 cells. Our results showed that knockdown of SKA1 expression led to reduced cell viability and increased apoptosis, as anticipated.

To date, there are very few reports on the role of SKA proteins in cancer formation and metastasis [21,22]. Clonogenic side population cells in some cancers, including multiple human myeloma and primary tumors, exhibit tumor-initiating characteristics. Gene expression analysis showed that clonogenic side population cells in cancers express genes involved in the cell cycle and mitosis (e.g., SKA1, CCNB1, CDC25C, CDC2, BIRC5, CENPE, AURKB, KIFs, TOP2A, ASPM) more strongly than non-side population cells [23]. In accordance with these reports, we found that SKA1 appears to be involved in oral carcinoma by regulating spindle checkpoint silencing and maintaining chromosome cohesion in mitosis. To the best of our knowledge, this study is the first to report that SKA1 expression is important in oral adenosquamous cancer cell proliferation. These findings will significantly enhance the current understanding of the role of the SKA complex in cancer cell proliferation. It is worth noting that the present data are only applicable to oral adenosquamous carcinomas. Thus, studies in other cell lines could be of interest. Moreover, future studies are needed to determine the precise mechanism underlying SKA1 regulation of oral adenosquamous cancer cell proliferation.

## Conclusions

This is the first report to show that SKA1 is important in the proliferation of oral adenosquamous cells. Thus, SKA1 silencing by RNAi might be a potential therapy for oral carcinoma.

## Methods

### Cell culture

CAL-27 and HEK 293 T cells were maintained in Dulbecco’s Modified Eagle’s Medium (DMEM) containing 10% Fetal Bovine Serum (FBS), and 4 mM L-glutamine, adjusted to contain 3.7 g/l sodium bicarbonate and 4.5 g/l glucose (Hyclone, Logan, UT). Cell cultures were maintained as a monolayer in 75 cm^2^ tissue-culture treated flasks (Bedford, MA) at 37°C in a humidified atmosphere of 5% CO_2_.

### Lentivirus production and infection of CAL-27 cells

The cDNA sequence of SKA1 was obtained from GenBank (accession number: NM_001039535). ShRNA for SKA1 (5′-CCG-GCC-TGA-CAC-AAA-GCT-CCT-AAA-TCT-CGA-GAT-TTA-GGA-GCT-TTG-TGT-CAG-GTT-3′) was inserted into the lentivirus expression plasmid pFH-L (Shanghai Hollybio, China). Non-silencing shRNA (5′-TTC-TCC-GAA-CGT-GTC-ACG-T-3′) was used as a control. In order to generate SKA1-shRNA and control lentivirus, lentiviral shRNA plasmids targeting SKA1 or a control were transfected into HEK293T cells using Lipofectamine 2000 (Invitrogen, Carlsbad, CA) together with the gag/pol packaging vector (pCMVΔR8.92, Shanghai Hollybio, China) and the pVSVG-1 encoding plasmid (Shanghai Hollybio) according to the manufacturer’s instructions. For lentivirus infection, CAL-27 cells were grown at a density of 5 × 10^4^ cells/well and infected with SKA1-shRNA lentivirus or control lentivirus at a MOI of 10.

### Determine the infection efficiency of SKA1-shRNA lentivirus

To determine the infection efficiency of SKA1-shRNA lentivirus, CAL-27 cells expressing GFP protein were observed by fluorescence microscopy (CKX41, Olympus, Tokyo-Japan).

For western blot analysis, CAL-27 cells were grown for 6 days. The cells were washed twice with ice-cold PBS, scraped, centrifuged, and lysed in RIPA buffer with PMSF (Beyotime Biotechnology, Jiangsu, China). Cell extracts were prepared on ice by ultrasonic disruption. After centrifugation at 12,000 × *g* for 15 min at 4°C, the supernatants were collected, and the protein concentrations were determined using the BCA Protein Assay Kit. Cell lysates were dissolved in 2x SDS sample buffer (100 mM Tris–HCl pH 6.8, 10 mM EDTA, 4% SDS, 10% glycine). Equal amounts of protein were resolved on 10% SDS-PAGE gels and electrophoretically transferred onto polyvinylidene fluoride membranes. Membranes were blocked in 5% skim milk and hybridized with primary antibodies (1:1000 dilution; SBA3500101, Sigma-Aldrich, St. Louis, MO, USA). After incubation with horseradish-peroxidase-conjugated secondary antibody (1:5000 dilution; sc-2004, Santa Cruz Biotechnology, Santa Cruz, CA, USA) at room temperature, immunoreactive proteins were detected using a Chemiluminescent ECL Assay Kit (Amersham Pharmacia Biotech, UK), according to the manufacturer’s instructions. GAPDH protein levels were used as a control to verify equal protein loading. Band intensities were analyzed with Quantity One analysis software (Bio-Rad, Hercules, CA, USA).

### MTT cell viability assay

In brief, both uninfected and infected CAL-27 cells (2.5 × 10^3^ cells/well) were seeded in 96-well plates. After 1, 2, 3, 4, or 5 days of infection, 100 μl MTT (5 mg/ml) was added, followed by incubation for another 4 h at 37°C. The reaction was stopped by replacing the MTT-containing medium with 100 μl acidic isopropanol (10% SDS, 5% isopropanol, 0.01 mol/l HCl), and the formazan salts were dissolved by gentle shaking for about 10 min at room temperature. For colorimetric analysis, the absorbance at 595 nm was recorded using a microplate reader (Bio-Rad). The optical of sample bands were analyzed with Quantity One analysis software (Bio-Rad, USA). Each assay was repeated at least three times. Relative cell viability was compared to the untreated (blank) group.

### Colony formation assay

Agar medium (0.8%) was prepared in 6-well plates. Uninfected and infected CAL-27 cells were trypsinized, centrifuged, resuspended in 0.4% agar medium (equal volumes of 0.8% agar and culture medium), and plated onto the top agar at 200 cells/well. Cell culture medium was changed regularly. After 13 days of culture, adherent cells were washed twice with PBS, and fixed in 4% paraformaldehyde for 30 min at room temperature. Colonies were stained with Giemsa solution for 72 h, washed with water, and air-dried. The colonies were counted under a fluorescence microscope.

### Cell cycle analysis

Uninfected and infected CAL-27 cells were seeded in 6-well culture plates at a density of 5 × 10^4^ cells/well, and incubated in complete medium to 70% confluence. The cells were harvested, washed twice with ice-cold PBS, and fixed in 75% cold ethanol at 4°C overnight. Propidium iodide (PI) staining of nuclei was used to monitor the cell cycle phases. The fluorescence of DNA-bound PI in cells was measured with a Cell Lab Quanta Beckman Coulter (Beckman Coulter, Miami, FL, USA), and data were analyzed using Multi-Cycle AV software (Phoenix Flow Systems, San Diego, CA, USA).

### Statistical analysis

PASW Statistics 18, Prism version 4.00 for Windows, and InStat 3.0 software were used for statistical data analyses. Numerical data were analyzed by one-way ANOVA. Data are represented as mean and standard error. Turkey’s test was used for multiple comparisons. P values less than 0.05 were considered statistically significant.

## Abbreviations

SKA1: Spindle and kinetochore associated complex subunit 1; RT-PCR: Real-time reverse transcription-polymerase chain reaction; siRNA: Small interfering double stranded RNA; shRNA: Short hairpin; LV-shNLK: Lentivirus/shRNA-NLK; LV-shCon: Letivirus/shRNA-Control.

## Competing interests

The authors declare that they have no competing interests.

## Authors’ contributions

BZ, and SWL have designed the project. In vitro determinations have been performed by BZ and KL Acquisitions of data: laboratory or clinical/literature search have been performed by HYC and SDP. Statistical study has been performed by LCJ. Drafting of the article and/or critical revision have been performed by BZ and YPW. Final approval and guarantor of manuscript have been performed by SWL. All authors read and approved the final manuscript.
